# A Decade of C3 Glomerulopathy—A Nationwide Cohort Study

**DOI:** 10.1016/j.ekir.2025.11.007

**Published:** 2025-11-12

**Authors:** Rick H. Overwijk, Fiona R. Kolbinger, Mark Eijgelsheim, Olaf M. Dekkers, Andreas Kronbichler, Ingeborg M. Bajema

**Affiliations:** 1 Division of Pathology, Department of Pathology and Medical Biology, University Medical Center Groningen and University of Groningen, Groningen, The Netherlands; 2 Weldon School of Biomedical Engineering, Purdue University, West Lafayette, Indiana, USA; 3 Division of Nephrology, Department of Internal Medicine, University Medical Center Groningen and University of Groningen, Groningen, The Netherlands; 4 Department of Clinical Epidemiology, Leiden University Medical Center, Leiden, The Netherlands; 5 Department of Clinical Epidemiology, Aarhus University and Aarhus University Hospital, Aarhus, Denmark; 6 Department of Endocrinology, Leiden University Medical Center, Leiden, The Netherlands; 7 Department of Internal Medicine IV, Nephrology and Hypertension, Medical University Innsbruck, Innsbruck, Austria; 8 Department of Health, Medicine and Caring Sciences, Linköping University, Linköping, Sweden

**Keywords:** C3 glomerulonephritis, C3 glomerulopathy, complement factor, dense deposit disease, postinfectious glomerulonephritis

## Abstract

**Introduction:**

C3 glomerulopathy (C3G) is a rare but devastating disease affecting children and adults. It frequently leads to end-stage kidney failure, and currently no specific treatment exists. C3G is used as a collective term for dense deposit disease (DDD) and C3 glomerulonephritis (C3GN) and is thought to sometimes occur in postinfectious settings (C3-PIGN). Currently, little is known about the incidence and distribution of subtypes in the population. We analyzed a large cohort of patients with C3G in the Netherlands regarding incidence and disease subtype distribution in relation to geographical factors and histopathological findings.

**Methods:**

A search in the Dutch Nationwide Pathology Databank (Palga) was performed to identify patients diagnosed with C3G from January 2014 until December 2023, subsequently ascertained by 2 independent observers. We assessed the correlation of disease subtypes with glomerular patterns, and the geographical distribution was charted.

**Results:**

The selection resulted in a cohort of 280 patients consisting of C3GN (*n* = 101), DDD (*n* = 39), unspecified C3G (*n* = 106), C3-PIGN (*n* = 29), and others (*n* = 5). The median age at biopsy diagnosis was 19 (range: 4–75) years for DDD and 54 (range: 2–86) years for C3GN, showing age distribution depends on C3G subtype (*P* < 0.001). DDD and C3G were associated with membranoproliferative pattern and C3-PIGN with endocapillary or exudative pattern.

**Conclusion:**

Our results show consistent assessment of kidney biopsies across the country and absence of geographical factors influencing disease development.

## Graphical abstract

**Figure:**
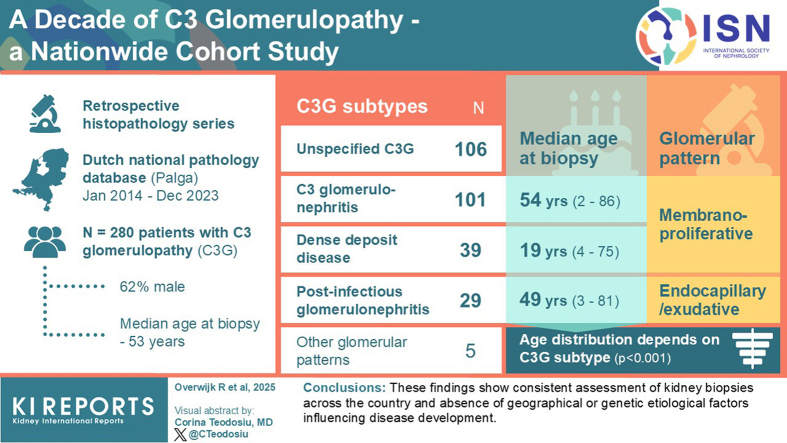

Approximately 10% of the world’s population is affected by chronic kidney disease.^1^ The Dutch population is no different in this aspect compared with other Western countries.^2^ Many inflammatory kidney diseases arise from deposits of Ig or complement factors in certain parts of the glomerulus. Immunofluorescence (IF) and immunohistochemical techniques in combination with electron microscopy (EM) are commonly used to determine the nature and microanatomical location of these deposits. C3G is a typical example of a disease defined by findings detected by IF and EM. C3G is a collective term that describes both C3GN and DDD, as per the 2013 consensus report.^3^ C3G is characterized by C3 deposits in the mesangium or subendothelial space in the case of C3GN and intramembranous deposits in the case of DDD. This is most likely caused by dysregulation of the alternative complement pathway.^4^^,^^5^ EM can reveal the exact microanatomical location, architecture, and electron density of these deposits. If they are highly osmiophilic and have a sausage-like appearance, the ultimate diagnosis is likely to be DDD. If the deposits are in the mesangial, subendothelial, or subepithelial spaces, and are less typically “dense,” the diagnosis is likely to be C3GN.^3^

C3G can manifest with a variety of clinical and histopathological presentations and occurs in both children and adults. Patients typically present with proteinuria, hematuria, and various degrees of kidney dysfunction. Younger patients usually develop these problems following an upper respiratory tract infection. Within 10 years from diagnosis, half of the patients develop kidney failure resulting in a need for kidney transplantation.^4^ In kidney biopsy, various patterns may occur, such as mesangioproliferative, membranoproliferative, or endocapillary (exudative) patterns.^6^ The variability of both the clinical and histological presentations can make C3G a challenging diagnosis.

In November 2013, the ISN published a report following a consensus meeting between experts in the fields of renal pathology, nephrology, complement biology, and complement therapeutics.^1^ Following this report, the term “C3 glomerulopathy” was used henceforth, because the earlier terminology was confusing and often not representative for describing the broad scope that this disease encompasses, including the different (histological) subtypes. It was recognized that renal pathology characterized by isolated or dominant C3 deposits was more heterogenous than previously thought.

Following the Mayo Clinic/Renal Pathology Society consensus report published in 2016,^3^ it is recommended that a kidney biopsy report at least covers, among other aspects, the pattern of glomerular injury based on light microscopy, as well as findings in IF and EM. It is generally recognized that C3G may occur in a (post) infectious setting, which could be explained by a dysregulation of the complement alternative pathway following kidney infection. In these cases, a potential preexisting complement dysregulation may result in ongoing complement activity after activation of the complement system by an infection, even if the infection has been successfully eliminated. Biopsies historically classified as postinfectious glomerulonephritis (PIGN) may nowadays be considered C3G.^7^ PIGN and C3G are probably part of a disease spectrum resulting in C3-dominant deposits,^4^ regardless of the cause of complement dysregulation, may be infectious, a primary (sometimes hereditary) complement disorder, or something different.

Currently, little is known about the exact etiology of C3G, and about its incidence and prevalence in the Dutch and general population. To our knowledge, studies on large C3G cohorts have not been published, presumably because of the relative rarity of the disease. Here, we describe a large cohort of C3G cases in the Netherlands, with patients diagnosed over the last 10 years, that is, since the appearance of the consensus report. We analyzed the geographical distribution of patients in relation to patient characteristics (age and biological sex as reported in the Palga databank), geographical factors (incidence, histological patterns, and C3G subtype distribution across different regions) and histopathological findings. With these data and assessments, we were be able to contribute to knowledge about aspects regarding prevalence and factors of influence related to this rare but severe disease.

## Methods

### Selection of Patient Reports and Inclusion Criteria

A search was performed in the Palga,^8^ to identify every kidney biopsy diagnosed with C3G in the past 10 years. January 1, 2014 was selected as the starting date, because the C3G consensus report published in October 2013 had introduced new nomenclature and criteria for the diagnosis and subtypes. Kidney biopsy reports were included if the conclusion of the report contained ≥1 of the following key terms (here represented in Dutch and without regard to punctuation marks): “C3-glomerulopathie, C3-glomerulopathy, C3-glomerulonefritis, C3-glomerulonephritis, DDD, membranoproliferatieve nefropathie, membranoproliferatieve glomerulonefritis, mesangiocapillaire glomerulonefritis, C3-depositie, or C3 complementfactor.” In case of multiple biopsy reports of 1 patient, for example, in case of kidney transplantation, only the first report was considered.

### Exclusion Criteria

Kidney biopsy reports were excluded from the analysis if the report met ≥ 1 of the following conditions: IF for C3 was completely negative; IF for C3 showed a nonnegative yet nondominant staining compared with other depositions (including but not limited to IgA, IgG, IgM, C1q, kappa, and lambda light chains); the primary biopsy was submitted and/or assessed before January 1, 2014; the biopsy material did not contain kidney tissue, or was not representative in another way; IF was not performed; the report was missing or incomplete. Sole external consultation and revision reports were excluded from the final dataset to avoid data duplication.

### Construction of the Data Set

After application of eligibility criteria, a concept dataset was constructed, whereafter screening was conducted by 2 independent observers. A consensus meeting was held to solve conflicting results. The final coded dataset contained the following variables: a randomly appointed patient number; sex; age at biopsy; date of biopsy; residential area grouped by cardinal point and province or province cluster; typical histopathological pattern; presence and assessment of EM; C3G subtype; whether the biopsy was from a native kidney or kidney transplant; and whether there was comorbidity, for example, diabetes with kidney disease or amyloidosis. The C3G subtypes were classified as follows: “DDD,” “C3GN,” “C3G without differentiation between DDD or C3GN,” “C3-PIGN,” “C3G associated with light chains or cryoglobulins.” The classification of the subtypes and the differentiation between DDD and C3GN was made based on the conclusion of the original report and central evaluation of the described findings by IF and EM. In the case of light chain disease, the description of IF showing nondominant staining of kappa and/or lambda light chains was indicative and the reports concerned were included as such.

### Statistical Analysis

Data were analyzed using IBM SPSS statistics data editor 28.^9^ Cross tabulations were used to determine a possible relation between the distribution of C3G subtypes, histological disease patterns, and 8 regions based on the provinces of The Netherlands, namely: Drenthe/Groningen/Friesland, Overijssel, Gelderland, Limburg, Noord-Brabant/Zeeland, Zuid-Holland, Noord-Holland, Flevoland/Utrecht, and unknown. The expected distributions per cardinal point, as well as per province or province cluster, were compared with actual distributions using chi-square tests. This method was used to compare primary C3G subtypes with primary glomerular histological patterns in the biopsies. Age distribution was compared between C3 subtypes using nonparametric analysis. For this, the expected age medians and distributions per C3G subtypes were compared with our measured results using a Kruskal-Wallis 1-way analysis of variance.

## Results

### Primary Results

**Figure 1:**
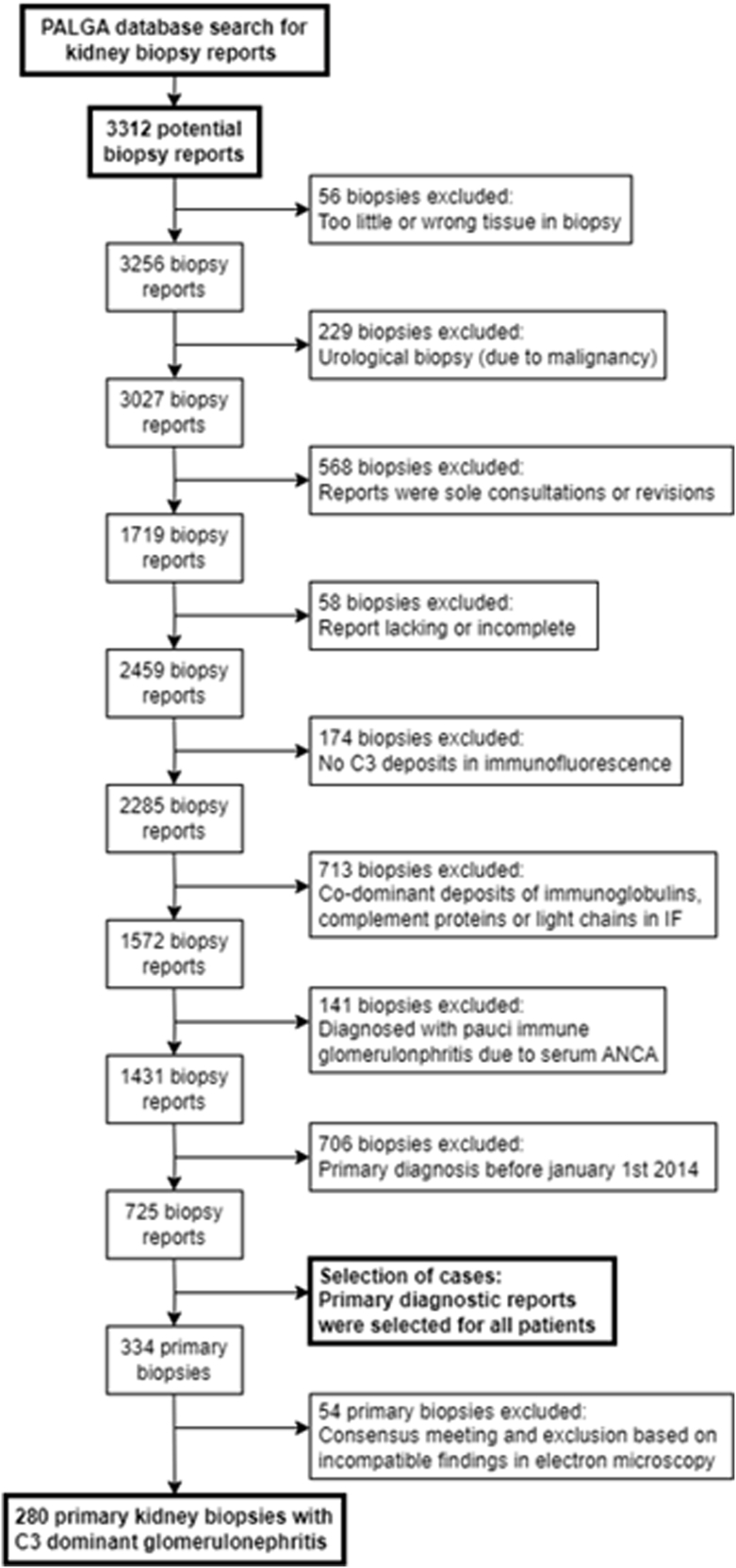
Flowchart of case selection showing number of excluded cases and applied criteria. ANCA, antineutrophil cytoplasmic autoantibody; IF, immunofluorescence; Palga, Dutch Nationwide Pathology Databank.

The primary Palga search resulted in a total of 3312 patient reports, which consisted of 1754 reports of first kidney biopsies and another 1558 reports of repeat biopsies. These included both native and allograft biopsies. The total number of patients was 1253. First, 56 reports were removed because of inadequate material. Then, 229 reports were excluded because the biopsy was taken with a clinical suspicion of malignancy. Another 568 reports were excluded because they were sole consultations or revisions of which the original biopsy reports could not be traced; they were excluded to avoid duplications. In addition, 58 biopsies lacked a (substantial) part of the report because of technical causes (e.g., resulting in incomplete text or missing IF or light microscopy), and were excluded. In 174 cases, there were no C3 deposits as described by the IF findings; 713 cases contained a description of a codominant, strong staining of Ig and/or light chains; all these cases were excluded. Moreover, 141 cases were excluded because the patients had positive serum antineutrophil cytoplasmic antibodies resulting in the diagnosis of pauci-immune glomerulonephritis. Lastly, 706 reports were removed because these patients were diagnosed before January 1, 2014. In total 725 reports were further examined. Of this group, only first biopsies of patients with multiple biopsies were selected, resulting in 334 biopsy reports of 325 native kidneys and 9 allografts. A consensus meeting resulted in the removal of 54 more biopsies. The final cohort contained 280 patients. Inherent to our search strategy, patients with Ig-mediated glomerulonephritis were, by definition, not included in this cohort. In Figure 1, we present the selection diagram.

### General Characteristics

**Figure 2:**
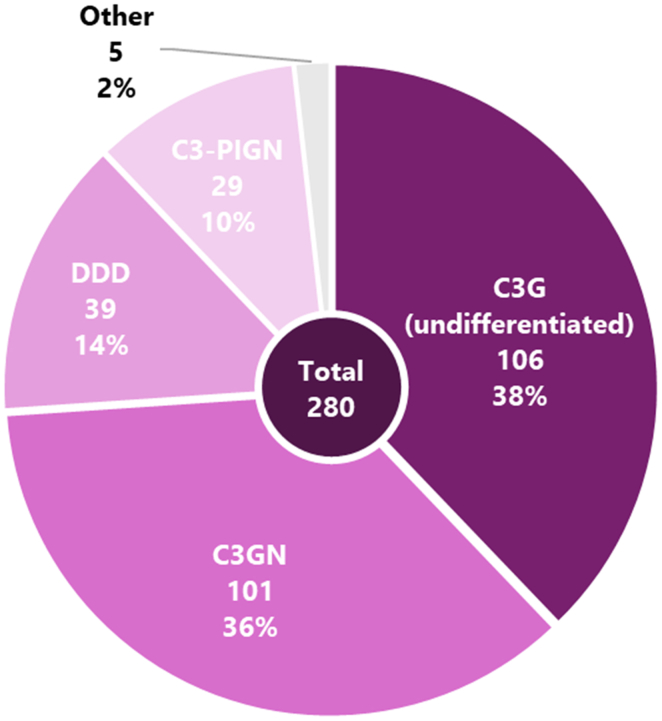
Pie chart showing the division in C3G subtypes based on the microscopy reports of included reports in percentages and number of cases. Total number of cases is 280. “Other” consists of cases in the context of light chain gammopathy and/or cryoglobulinemia. C3G, C3 glomerulopathy; C3GN, C3 glomerulonephritis; DDD, dense deposit disease.

**Figure 3:**
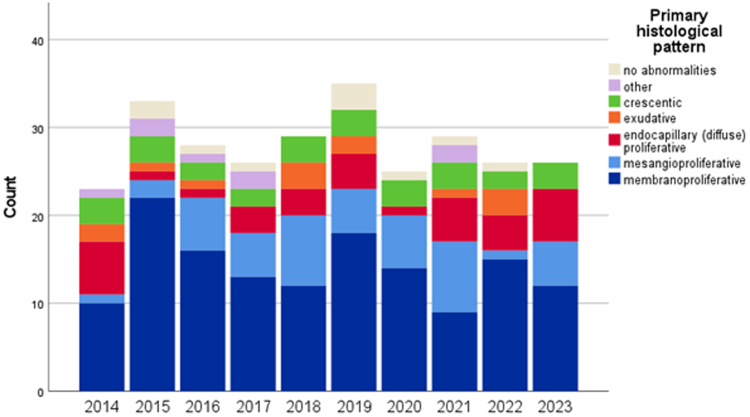
Stacked bar graph showing incidence of C3 glomerulopathy per year from 2014 until 2023, in this figure subdivided by histological pattern. It is noticeable that the membranoproliferative pattern is consistently found to be the most common glomerular disease pattern, suggesting consistent biopsy assessment.

Of the total 280 patients, 175 were male (62.5%). The age ranged from 1 to 86 years with a median of 53 years. A total of 101 patients were classified as C3GN (36.1%) (Figure 2), 39 were classified as DDD (13.9%), and 106 were classified as C3G without differentiation between DDD and C3GN (37.9%), mostly because of unavailable EM. Another 29 patients (10.0%) were found to have C3-PIGN and in 5 cases there was an association with monoclonal light chains (found in the biopsy with IF or immunohistochemistry) or cryoglobulins (found with testing patient’s serum, only when mentioned in the biopsy report). When excluding the C3-PIGN and light chain/cryoglobulin subtypes, 246 patients with “true” C3G, according to the most recent diagnostic criteria, remained. When calculating the average incidence in the last 10 years, the average figures of the Dutch population from January 2014 to December 2023 was used, namely 17.25 million people. This resulted in an average incidence of C3G in the Netherlands of 1.43 patients per million/yr. From 2014 onward, there was a relatively consistent number of newly diagnosed cases (Table 1 and Figure 3).

**Table 1:** Table with incidence per year, showing consistent occurrence of C3G across time in the research period

Year	2014	2015	2016	2017	2018	2019	2020	2021	2022	2023		C3G + PI	C3G − PI
*n*	23	33	28	26	29	35	25	29	26	26	Totals	280	246
Population (million)	16.83	16.9	16.98	17.08	17.18	17.28	17.41	17.48	17.59	17.81	Mean	17.25	17.25
Incidence (*n*/million)	1.37	1.95	1.65	1.52	1.69	2.03	1.44	1.66	1.48	1.46	Mean	1.62 (± 0.02)	1.43 (± 0.03)

C3G + PI, incidence including postinfectious C3G; C3G − PI, incidence excluding postinfectious C3G; *n*, number of cases; pop., population in millions.

### Relation With Geographical Location

**Figure 4:**
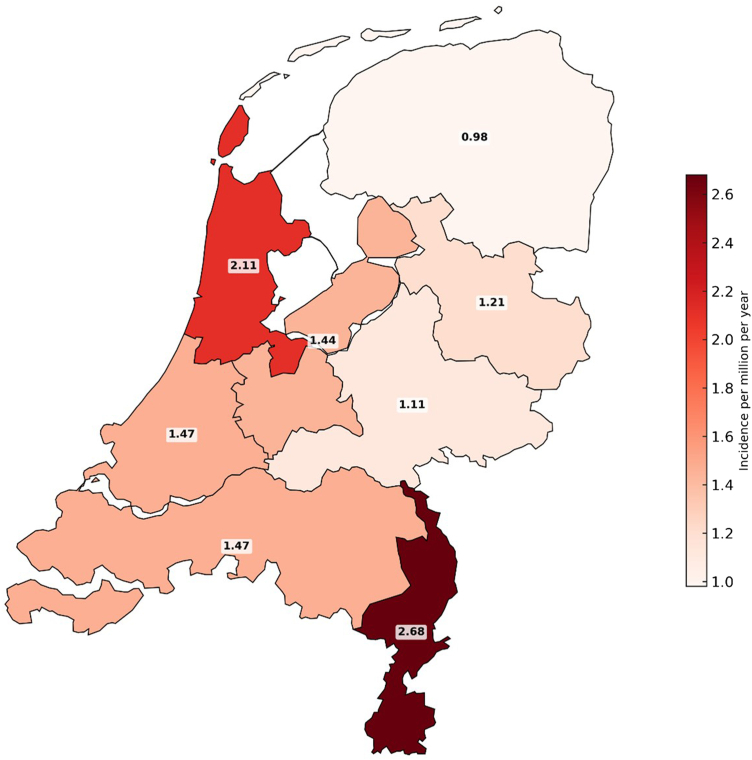
Map of the Netherlands showing the incidence rates per province (cluster), expressed in number of cases per million per year during the research period of January of 2024 to December of 2023.

**Figure 5:**
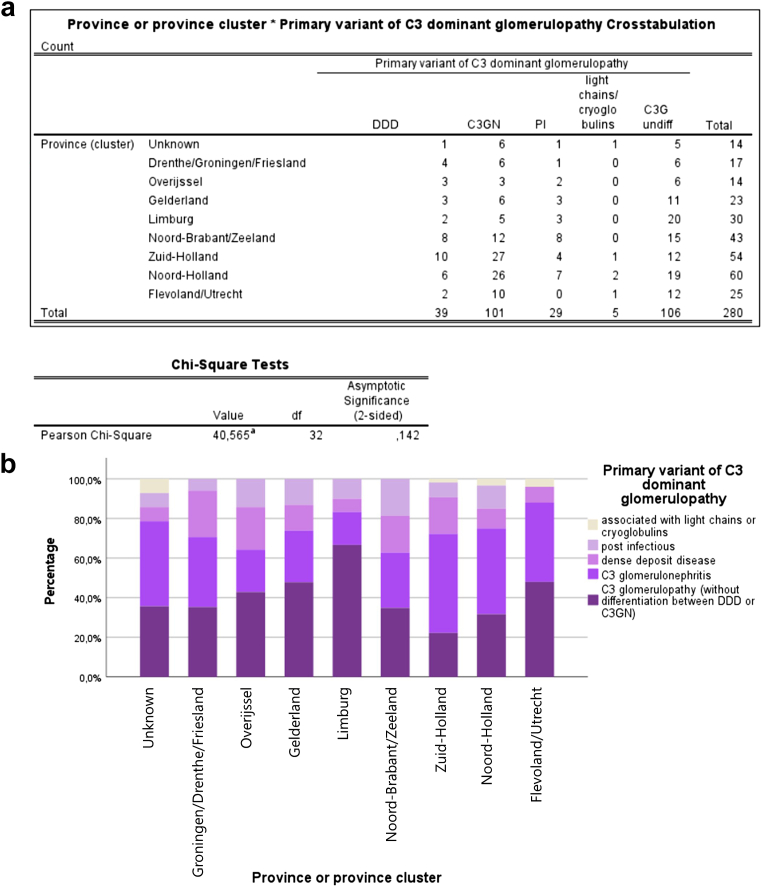
Chi-square test (a) and stacked bar graph (b) showing no statistical significant variations of C3G subtype distribution between province (cluster). Chi-square test with *P*-value > 0.05. C3G, C3 glomerulopathy; C3GN, C3 glomerulonephritis; DDD, dense deposit disease.

Most patients with C3G were diagnosed in the western provinces of the Netherlands, namely 60 in North Holland and 54 in South Holland. In the southern provinces, 43 cases were diagnosed in North Brabant and Zeeland combined, as well as 30 in Limburg. In the middle and eastern provinces, 25 were diagnosed in Flevoland and Utrecht, 23 in Gelderland, and 14 in Overijssel. The northern provinces combined (Drenthe, Groningen, and Friesland) included 17 patients. For 14 patients, the postal code was unknown. When the overall C3G subtype distribution was compared between province or province cluster, no major variations in the distribution patterns was observed (*P* = 0.142) (Figures 4 and 5). Comparison of the incidence rates and their 95% confidence intervals (based on calculated standard errors) showed no marked differences in incidence per province or province cluster, indicating that even though numbers in this cohort are small, incidence rates do not give rise to the assumption that the distribution of patients over the Netherlands would be uneven.

### Age Distribution Across C3G Subtypes

**Figure 6:**
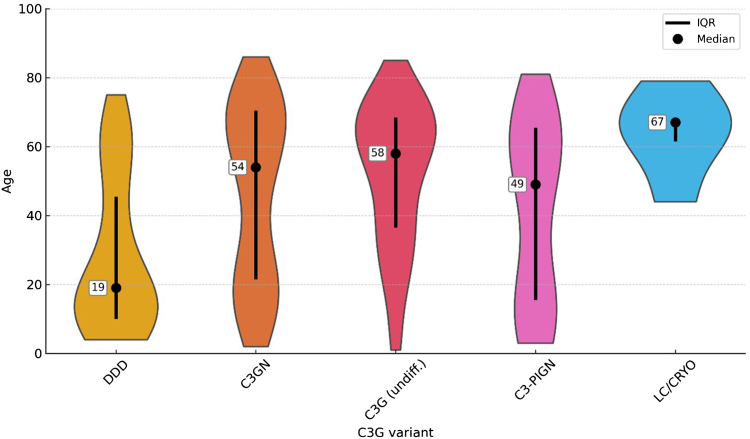
Violin plots showing age distribution and median for the C3G subtypes, most noticeably, the lower median age and spread of DDD compared with other subtypes. C3G, C3 glomerulopathy; C3GN, C3 glomerulonephritis; C3-PIGN, C3–postinfectious glomerulonephritis; DDD, dense deposit disease; IQR, interquartile range; LC/CRYO, C3G cases associated with light chain gammopathy and/or cryoglobulins.

Analysis showed a difference in age distributions between DDD and the other subtypes (Figure 6). The age range of DDD was 4 to 75, with a median of 19 years. In comparison, the median age of C3GN was 54 (range: 2–86) years, the median age of the undifferentiated group was 58 (range: 1–85) years and the median age of C3-PIGN was 49 (range: 3–81) years.

### Relation Between Histological Glomerular Patterns and C3G Subtypes

**Figure 7:**
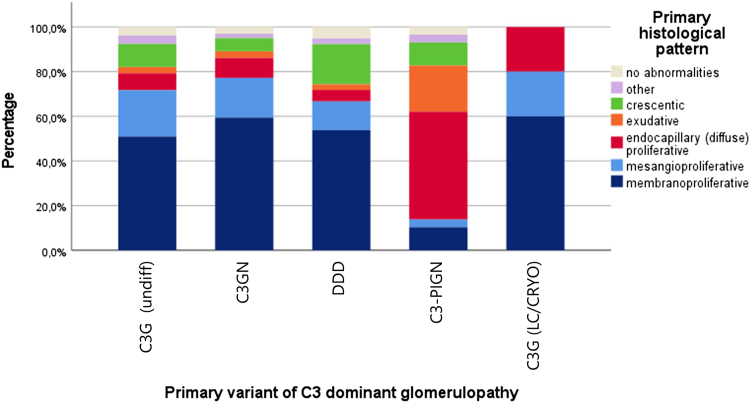
Stacked bar graph showing the different histological disease patterns that were found, grouped per C3G variant. This clearly shows that the proportion of kidney biopsies with a membranoproliferative pattern was higher in those with undifferentiated C3G, C3GN, DDD, and C3G in association with light chain gammopathy or cryoglobulins. For a mesangioproliferative pattern this was also the case. Moreover, it is shown that relatively more C3-PIGN cases showed an endocapillary pattern. It is also worth noting that in most subgroups, a small subset of biopsies showed a crescentic glomerular pattern. C3G, C3 glomerulopathy; C3GN, C3 glomerulonephritis; C3-PIGN, C3-post-infectious glomerulonephritis; DDD, dense deposit disease.

On multivariate analysis, we identified a relation between C3G (undifferentiated), C3GN and DDD, and a primary membranoproliferative glomerular pattern, although all other histopathological patterns were found in all other subtypes (Figure 7). Of the 29 patients with C3-PIGN, most cases showed an endocapillary or exudative pattern. In all subtypes, excluding light chains/cryoglobulins, a subset of 27 cases (9.6%) showed a crescentic glomerulonephritis.

### Distribution of Histological Glomerular Patterns per Province (Cluster)

**Figure 8:**
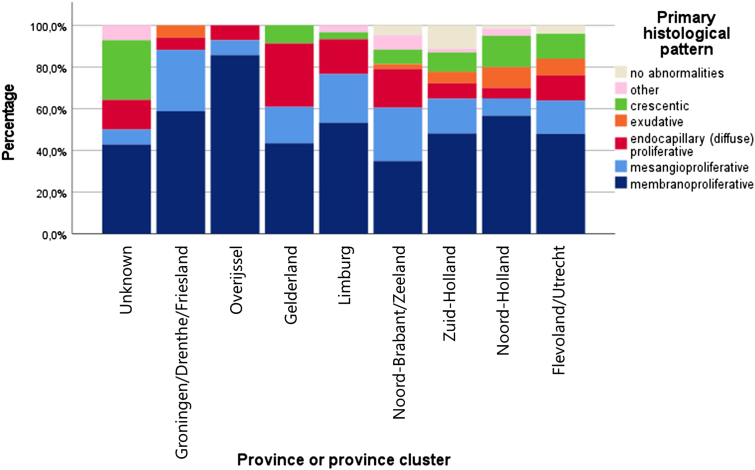
Bar graph showing the distribution of histological glomerular pattern per province (cluster). This shows a dominant membranoproliferative pattern in all research regions.

The distribution of the histological patterns based on light microscopy by province cluster showed a significant variation using the chi-square test (*P* = 0.035; Figure 8). This indicates that the distribution across regions of the Netherlands is not entirely homogenous. However, from the graph, we can conclude that the most common histological pattern in all regions is a membranoproliferative glomerulonephritis. Most regions show a second common pattern of mesangioproliferative glomerulonephritis.

## Discussion

In this study, the largest cohort of C3G known today, as we are aware of, was constructed, based on pathology report data in the Netherlands provided by Palga, encompassing 280 patients with a biopsy diagnosis established between January 2014 and December 2023. We present the geographical and histopathological data of this cohort which contains a first diagnosis of C3G in native kidneys covering the 10-year period after the establishment of new nomenclature and diagnostic criteria. The Dutch Palga system provided us with the unique opportunity to establish this group of 280 patients. To our knowledge, there is only 1 other study that performed a nationwide investigation to evaluate the prevalence and distribution of patients with C3G; Candela *et al.*^10^ published characteristics of all patients with biopsy-proven C3G in French Polynesia. Their point prevalence of patients with C3G was approximately 3 times higher than in our population—which may have been influenced by the homogeneity of the environment and the genetic background of this relative small population (∼ 300.000 inhabitants). Alternatively, the reluctance to perform a biopsy may have been different in French Polynesia and the Netherlands—it is possible that in French Polynesia patients with a milder disease presentation were biopsied more frequently. The male predominance and the median age are the same for both cohorts.

There is no indication for regional clustering of C3G in The Netherlands. Although C3G has an overall higher incidence in the Western provinces, this is likely because of the higher population density in these areas. In addition, C3G subtypes and histological patterns are evenly distributed across The Netherlands. These results give no rise to the assumption of clustering in terms of families or relatives sharing a possible genetic background that would lead to an increased prevalence in certain previously undefined districts. However, the data suggest that pathologists in the Netherlands make equally consistent diagnoses within the C3G spectrum.

The starting point for our cohort was based on the nomenclature consensus described above. Earlier terminology could frequently not be used satisfactory to describe the various histopathological patterns that could be found. DDD would be described as “membranoproliferative glomerulonephritis (MPGN, sometimes also called mesangiocapillary glomerulonephritis) type II,” or “MPGN type I with isolated C3 deposits.” However it has been known for some time that most DDD cases show a different (inflammatory) glomerular pattern than MPGN.^6^ In our cohort, there was a predominance of the MPGN pattern in all C3G subtypes followed by the mesangioproliferative pattern in line with the findings of other studies.^11^ Only C3-PIGN had a closer correlation with endocapillary and exudative patterns as expected; because we relied on this cohort on the diagnosis given in the original report, we cannot determine the exact interaction between the C3-PIGN diagnosis and its accompanying histological pattern, thereby suggesting that endocapillary and exudative patterns would probably enhance the chance of the diagnosis becoming C3-PIGN. Nevertheless, the variability of subtypes in each category was noticeable with multiple cases identified to have different histological patterns, and some to have no histological abnormalities at all. This means that based on histological pattern alone, the diagnosis of C3G cannot be made confidently. Our results therefore emphasize that in current medical practice, the combination of IF and EM are essential to diagnose and subtype C3G correctly, which is necessary to propose optimal treatment strategies and provide useful prognostic information.

Recent studies on C3G have focused mainly on clinical characteristics, new therapy regimens, and disease outcome based on a variety of biomarkers with little attention to the histopathological pattern of C3G.^12^^,^^13^ Whereas it is known that C3G can present with a tremendous variability of patterns,^4^ studies focusing on outcome in relation to histopathology have rather focused on more general patterns such as individual histological parameters,^14^ chronicity scores,^15^ a histologic scoring index,^16^ or even by developing a nomogram.^17^ For research purposes, these set-ups are certainly of interest, but they do rely upon a previously established, biopsy-proven diagnosis which can only be obtained by optimal usage of IF and EM in combination with the pattern seen by light microscopy.

For C3G in particular, providing sufficient clinical data to the pathologist is important, for example C3 serum levels or a history of infectious diseases, as a reminder that C3G should be part of the differential diagnosis, and access to and sufficient knowledge of IF and EM are of vital importance^18^—thereby guiding the choice of tissue material preserved for these 2 additional techniques.

The drawbacks of this study are that, until now, the cohort is based on written reports, with a central review of the biopsy still lacking. However, because kidney biopsies in The Netherlands are usually reported by expert kidney pathologists, the quality of the reports was high and comparable in the group of patients that we established. Moreover, at this moment, neither detailed follow-up data on patients’ clinical course, nor information about the genetic background is available. This clinical data, such as serological evaluation (e.g., cryoglobulinemia or monoclonal light chain gammopathy) or course of disease (e.g., precursory infection), was not uniformly available for all cases, only if mentioned in the report by the pathologist. We realize this could have resulted in an underestimation of alternative diagnoses with a C3 dominant glomerular disease patterns.

### Future Perspectives

Our study presents the largest cohort of patients with C3G described so far in the literature; however, drawbacks of the study are the reliance on written reports and the lack of detailed follow-up data. Our future perspectives include a central review of the biopsies and collection of detailed clinical data. We intend to focus in the near future on studies that will use the cohort as a stepping stone for investigating the predictive value of kidney biopsy in regard to clinical outcomes; to analyze the quality of the biopsy report and of EM and IF investigations in more detail; and to work on an artificial intelligence algorithm by which the diagnosis of the various subtypes of C3G can be ameliorated.

## Disclosure

AK received grant support from CSL Vifor and Otsuka; consultancy fees from Amgen, AstraZeneca, Boehringer Ingelheim, CSL Vifor, Delta4, GlaxoSmithKline, Novartis, Novo Nordisk, Otsuka, Roche, Sobi, and Walden Biosciences; and serves as an Associate Editor of Glomerular Diseases and as an Editor of Nephrology Dialysis Transplantation. FK is supported by the German Cancer Research Center (CoBot 2.0), the Joachim Herz Foundation (Add-On Fellowship for Interdisciplinary Life Science), and the German Research Foundation (Deutsche Forschungsgemeinschaft, DFG) as part of Germany’s Excellence Strategy (EXC 2050/1, Project ID 390696704) within the Cluster of Excellence “Centre for Tactile Internet with Human-in-the-Loop” (CeTI) of the Dresden University of Technology. Furthermore, FK receives support from the Indiana Clinical and Translational Sciences Institute funded, in part, by Grant Number UM1TR004402 from the National Institutes of Health, National Center for Advancing Translational Sciences, Clinical and Translational Sciences Award. ME declared advisory roles for Novartis and ViforPharma (fee to institution). All the other authors declared no competing interests.

## Supplementary Material

Supplementary File (PDF) STROBE Checklist.

## References

[1] Kovesdy C.P. (2022). Epidemiology of chronic kidney disease: an update 2022. Kidney Int Suppl (2011).

[2] Flinterman L., Heins M., Leemrijse C., Korevaar J., Schermer T. Vroege opsporing chronische nierschade, Nederlands Instituut voor Onderzoek van de Gezondheidszorg (Nivel). https://www.nivel.nl/sites/default/files/bestanden/Vroege_opsporing_chronische_nierschade.pdf.

[3] Pickering M.C., D’agati V.D., Nester C.M. (2013). C3 glomerulopathy: consensus report. Kidney Int.

[4] Mehdi A., Taliercio J.J. (2023). C3 glomerulopathy. Cleve Clin J Med.

[5] Roquigny J., Meuleman M.S., El S.C. (2025). Acquired and genetic drivers of C3 and C5 convertase dysregulation in C3 glomerulopathy and immunoglobulin-associated MPGN. Nephrol Dial Transplant.

[6] Koopman J.J.E., Vries D., Bajema I.M., APJ, Bajema I.M. (2021). C3 glomerulopathy. Nephrol Dial Transplant.

[7] Al-Ghaithi B., Chanchlani R., Riedl M., Thorner P., Licht C. (2016). C3 glomerulopathy and post-infectious glomerulonephritis define a disease spectrum. Pediatr Nephrol.

[8] Casparie M., Tiebosch A.T.M.G., Burger G. (2007). Pathology databanking and biobanking in the Netherlands, a central role for PALGA, the Nationwide Histopathology and Cytopathology Data Network and Archive. Anal Cell Pathol.

[9] SPSS IBM (2022). Statistics for Windows [Computer software]. Version 28.0.

[10] Candela N., Benichou N., Lefebvre M. (2024). C3 glomerulopathy is highly prevalent in French Polynesia. J Transl Autoimmun.

[11] Medjeral-Thomas N.R., O’Shaughnessy M.M., O’Regan J.A. (2014). C3 glomerulopathy: clinicopathologic features and predictors of outcome. Clin Js Am Soc Nephrol.

[12] Ravindran A., Fervenza F.C., Smith R.J.H., De Vriese A.S., Sethi S. (2018). C3 glomerulopathy: ten years’ experience at Mayo Clinic. Mayo Clin Proc.

[13] Chauvet S., Hauer J.J., Petitprez F. (2022). Results from a nationwide retrospective cohort measure the impact of C3 and soluble C5b-9 levels on kidney outcomes in C3 glomerulopathy. Kidney Int.

[14] Lomax-Browne H.J., Medjeral-Thomas N.R., Barbour S.J. (2022). Association of histologic parameters with outcome in C3 glomerulopathy and idiopathic immunoglobulin-associated membranoproliferative glomerulonephritis. Clin J Am Soc Nephrol.

[15] Caravaca-Fontán F., Cavero T., Díaz-Encarnación M. (2023). Clinical profiles and patterns of kidney disease progression in C3 glomerulopathy. Kidney360.

[16] Bomback A.S., Santoriello D., Avasare R.S. (2018). C3 glomerulonephritis and dense deposit disease share a similar disease course in a large United States cohort of patients with C3 glomerulopathy. Kidney Int.

[17] Caravaca-Fontán F., Rivero M., Cavero T. (2022). Development and validation of a nomogram to predict kidney survival at baseline in patients with C3 glomerulopathy. Clin Kidney J.

[18] Hou J., Ren K.Y.M., Haas M. (2022). C3 glomerulopathy: a review with emphasis on ultrastructural features. Glomerular Dis.

